# Rabi splitting and optical Kerr nonlinearity of quantum dot mediated by Majorana fermions

**DOI:** 10.1038/s41598-018-35680-1

**Published:** 2018-12-05

**Authors:** Hua-Jun Chen, Hong-Wei Wu

**Affiliations:** School of Mechanics and Photoelectric Physics, Anhui University of Science and Technology, Huainan Anhui, 232001 China

## Abstract

Majorana fermions (MFs), due to their significance in fundamental physics and potential applications in topological quantum computation based on solid-state devices, have drawn widespread attention. Here, we design a hybrid semiconductor/superconductor ring (S/SR) device and put forward an optical method for detecting a pair of MFs in the S/SR device with a quantum dot (QD) with the popular optical pump-probe scheme, which is very different from the present electrical method only considering one nearby MF coupled to the QD. The symmetric and unsymmetric Rabi splitting and optical Kerr nonlinear effect of QD mediated by MFs are investigated under uncoupled and coupled majorana modes, respectively. The coherent optical spectra indicate that a pair of MFs coupled to the QD induced remarkable splitting under different parameters regime and the physical origin of these phenomena are elaborated detailedly. Due to QD-MFs coupling, the probe absorption spectra present the phenomenon of Majorana modes induced transparency (MMIT) which will induce remarkable phenomena of slow light. The coherent optical spectra afford a potential supplement for probing MFs and support Majorana fermions-based topological quantum computation.

## Introduction

Majorana fermions (MFs) are exotic particles whose antiparticle is itself $\gamma ={\gamma }^{\dagger }$ obeying non-Abelian statistics. Therefore MFs can realize subsequent potential applications in topological quantum computation and quantum information processing^1^. Despite proposed initially to investigate neutrinos, one has discovered the analogous Majorana zero modes in condensed matter systems^2^. Currently, Majorana signature have been observed experimentally in various hybrid systems including hybrid semiconducting nanowire (atomic chains)/superconductor structure^3–7^, iron-based superconductor^8^, topological structure^9,10^, and quantum anomalous Hall insulator–superconductor structure^11^. For detecting MFs, several typical means have also been presented experimentally, such as zero-bias peaks (ZBPs) in tunneling spectroscopy^3–7^, fractional a.c. Josephson effect^12^, Coulomb blockade spectroscopy experiment^9^, and spin-polarized scanning tunneling microscopy^13^. We notice that most of the recent theories and experiments for researching and detecting MFs proposed and carried out focus on electrical scheme, and other effective and alternative methods, for example, all-optical means for probing Majorana bound states have received less attention.

**Figure 1:**
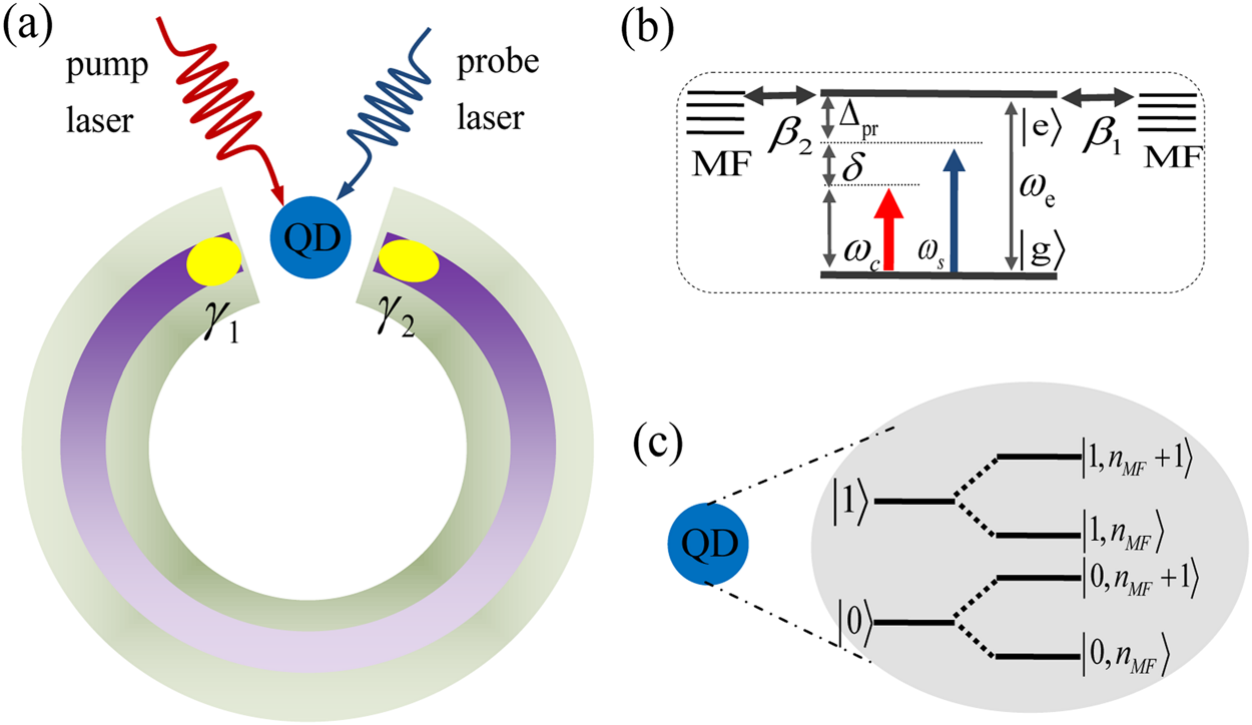
(**a**) shows a semiconducting ring with strong spin-orbit interaction in an external aligned parallel magnetic field B is placed on the surface of a superconducting ring different from the previous work^3,6^, and a couple of Majorana fermions (MFs) emerge at the end of semiconducting ring. A quantum dot (QD) driven by the pump-probe technology is introduced to probe a pair of MFs signature. When a couple of MFs appear in the semiconducting ring and interact with the QD, Majorana signatures will be brought out with the coherent optical spectra of the QD. (**b**) The energy-level of the QD interact with two MFs. (**c**) We consider the QD as a TLS rather than only owns one resonant level with single spin state in the electrical detection means^23–27^, and the QD coupled to a couple of MFs will induce the coupled states $|\mathrm{0,}\,{n}_{MF}\rangle$, $|\mathrm{0,}\,{n}_{MF}+1\rangle$, $|\mathrm{1,}\,{n}_{MF}\rangle$ and $|\mathrm{1,}\,{n}_{MF}+1\rangle$ (*n*_*MF*_ denotes MFs number states).

On the other hand, nanostructures such as quantum dots (QDs) have been obtained remarkable progress in modern nano-science and nano-technology in recent years^14^. QD, consiered as a simple stationary “artificial atom” with well optical property^15^, lays the foundation for numerous potential applications^16^, where the significant applications including entanglement concentration, logic gate construction and entangled state preparation with optical Kerr nonlinear effect have drawn widespread attention^14,17,18^. The optical Kerr nonlinear effect manifested by the third-order optical Kerr nonlinearity of QD, which is essential for light-controlled phase and refractive index modulation in various fields such as optical telecommunications, optical data storage, and information processing^14,19^. If coupling the QD to metallic nanoparticle (MNP)^20,21^, the plasmon resonance on MNP will amplify the local field near the QD and thereby intensify the mechanically induced optical Kerr nonlinearity, which will realize several orders of magnitude in the optical Kerr effect larger than in intrinsic Si and other hybrid systems^22^. Further, the detection of MFs with the QD have been proposed theoretically^23–27^, and most recently Majorana bound states have been investigated at the end of epitaxial hybrid Sm-S nanowires using tunneling spectroscopy through QDs^28^. However, in these schemes, QD is consider as only one resonant level with single spin state^23–27^, and we will consider the QD as a two-level system (TLS) (as shown in Fig. 1(b)) which is different from previous theoretical schemes for detecting MFs.

In this work, rather than previous schemes for probing MFs in electrical domain, firstly, we design a hybrid semiconductor/superconductor ring (S/SR) device as shown in Fig. 1(a), and the QD is driven by the optical pump-probe technology^29^ for detecting a couple of MFs emerging in the ends of the semiconducting nanowire ring under in a magnetic field. Due to the QD has no direct contact with the S/SR device, the scheme is immune to the disturbance of other signals in detecting of MFs with the optical means. Once a couple of MFs appear at the end of the nanowire ring and have interaction with the QD, the signatures of MFs will be brought out with the coherent optical response. Secondly, we further investigate the Rabi splitting and optical Kerr nonlinear effect of QD mediated by MFs. The probe absorption spectra present symmetrical and asymmetrical Rabi splitting under uncoupled MFs modes (ϵ_*MF*_ = 0) and coupled MFs modes (ϵ_*MF*_ ≠ 0), respectively. When a pair of MFs couple to the QD, the Rabi splitting is enhanced, which depends on the coupling strength between a pair of MFs and the QD. With adjusting the exciton resonant detuning and Majorana resonant detuning, the probe absorption intensity and the peak width of splitting are enhanced significantly. The optical Kerr nonlinear phenomena are also investigated under different parameter regions, and the peak width of splitting in the optical Kerr spectrum depends on the coupling strength and resonant detuning.

## Results

### The Model and Hamiltonian

Figure 1 shows a QD coupled to a pair of MFs emerging in the hybrid S/SR device. We consider the QD as a TLS which include the ground state $|g\rangle$ and the single exciton state $|e\rangle$^30,31^, and its Hamiltonian can be described as *H*_*QD*_ = *ℏω*_*e*_*S*^*z*^, where *ω*_*e*_ is the exciton frequency. We introduce the pseudo-spin operators *S*^*z*^ and *S*^±^ to describe the QD, which has the commutation relations [*S*^*z*^, *S*^±^] = ±*S*^±^ and [*S*^+^, *S*^−^] = 2*S*^*z*^. In order to describe MFs, we also introduce an operator *γ*_*M*_ with relation ${\gamma }_{M}^{\dagger }={\gamma }_{M}$ $({\gamma }_{M}^{2}=1)$ due to MFs are their own antiparticle. Then the coupling Hamiltonian of QD and a pair of MFs *γ*_*M*1_ and *γ*_*M*2_ is^23–27^.

1 $${H}_{int}=i{\epsilon }_{MF}{\gamma }_{M1}{\gamma }_{M2}/2+i\hslash {\beta }_{1}({S}^{-}-{S}^{+}){\gamma }_{M1}+i\hslash {\beta }_{2}({S}^{-}-{S}^{+}){\gamma }_{M2},$$

the above Hamiltonian includes three parts, the first one is *i*ϵ_*MF*_*γ*_*M*1_*γ*_*M*2_/2 which gives the energy of MFs (${\epsilon }_{MF}=\hslash {\omega }_{MF} \sim {e}^{-l/\xi }$, *ω*_*MF*_ is the Majorana frequency, *l* is the semiconductor length, and *ξ* is the superconductor coherent length). If *l* is long enough, ϵ_*MF*_ will be close to zero. We will investigate the two conditions detailedly in the following section and define them as the coupled- (ϵ_*MF*_ ≠ 0) and uncoupled-Majorana modes (ϵ_*MF*_ = 0). The second and the third term describe a pair MFs coupled to the QD with the coupling strengths *β*_1_ and *β*_2_, and the coupling strengths relate to the distance between the QD and S/SR device. For simplicity, the Majorana representation *γ*_*M*_ is transformed to the usual fermion operator with the transformation of ${\gamma }_{M1}={f}_{M}^{\dagger }+{f}_{M}$ and ${\gamma }_{M2}=i({f}_{M}^{\dagger }-{f}_{M})$, where ${f}_{M}^{\dagger }$ (*f*_*M*_) is the fermion creation (annihilation) operator. Using rotating wave approximation^32^, Eq. (1) can be rewritten as

2 $${H}_{MFs-QD}={\epsilon }_{MF}({f}_{M}^{\dagger }{f}_{M}-1/\mathrm{2)}+i\hslash {\beta }_{1}({S}^{-}{f}_{M}^{\dagger }-{S}^{+}{f}_{M})-\hslash {\beta }_{2}({S}^{-}{f}_{M}^{\dagger }+{S}^{+}{f}_{M}\mathrm{).}$$

We have neglected the non-conservation term of energy incluing $i\hslash {\beta }_{1}({S}^{-}{f}_{M}-{S}^{+}{f}_{M}^{+})$ and $\hslash {\beta }_{2}({S}^{-}{f}_{M}+{S}^{+}{f}_{M}^{+})$, and numerical results indicate that the influence of them are too weak to be considered in the theoretical treatment.

The Hamiltonian of ${H}_{QD-F}=-\,\mu {E}_{c}({S}^{+}{e}^{-i{\omega }_{c}t}+{S}^{-}{e}^{i{\omega }_{c}t})-\mu {E}_{s}({S}^{+}{e}^{-i{\omega }_{s}t}+{S}^{-}{e}^{i{\omega }_{s}t})$ indicates a strong pump laser and a weak probe laser simultaneously irradiating to the QD^33^ and has a interaction with the QD, in which *μ* is the electric dipole moment of the QD, *ω*_*c*_ and *ω*_*s*_ with light intensity *E*_*c*_ and *E*_*s*_ are the frequencies of the pump laser and probe laser fields, respectively. We use the rotating frame of the pump laser frequency *ω*_*c*_, and then obtain the full Hamiltonian of our hybrid system^34^

3 $$\begin{array}{c}H=\hslash {{\rm{\Delta }}}_{c}{S}^{z}+\hslash {{\rm{\Delta }}}_{MF}({f}_{M}^{\dagger }{f}_{M}-1/\mathrm{2)}+i\hslash {\beta }_{1}({S}^{-}{f}_{M}^{\dagger }-{S}^{+}{f}_{M})-\hslash {\beta }_{2}({S}^{-}{f}_{M}^{\dagger }+{S}^{+}{f}_{M})\\ \,-\hslash {{\rm{\Omega }}}_{c}({S}^{+}+{S}^{-})-\mu {E}_{s}({S}^{+}{e}^{-i\delta t}+{S}^{-}{e}^{i\delta t}),\end{array}$$

where Δ_*c*_ = *ω*_*e*_ − *ω*_*c*_ is the exciton-pump field detuning, Δ_*MF*_ = *ω*_*MF*_ − *ω*_*c*_ is the Majorana-pump field detuning, and *δ* = *ω*_*s*_ − *ω*_*c*_ is the probe-pump detuning. Ω_*c*_ = *μE*_*c*_/ℏ indicates the Rabi frequency of the pump field.

### The Quantum Langevin Equations (QLEs)

We use the Heisenberg equation of motion *i*ℏ∂_*t*_*ρ* = [*ρ*, *H*] (*ρ* = *S*^*z*^, *S*^−^, *f*_*M*_) and introduce corresponding damping and noise operators, we obtain the QLEs as^35^:

4 $${\partial }_{t}{S}^{z}=-\,{{\rm{\Gamma }}}_{1}({S}^{z}+1/\mathrm{2)}-{\beta }_{1}({S}^{-}{f}_{M}^{\dagger }+{S}^{+}{f}_{M})-i{\beta }_{2}({S}^{-}{f}_{M}^{\dagger }+{S}^{+}{f}_{M})+i{{\rm{\Omega }}}_{c}({S}^{+}-{S}^{-})\,+(i\mu {E}_{s}/\hslash )({S}^{+}{e}^{-i\delta t}-h\mathrm{.}c),$$

5 $${\partial }_{t}{S}^{-}=-(i{{\rm{\Delta }}}_{c}+{{\rm{\Gamma }}}_{2}){S}^{-}+\mathrm{2(}{\beta }_{1}-i{\beta }_{2}){S}^{z}{f}_{M}-2i{{\rm{\Omega }}}_{c}{S}^{z}-2i\mu {E}_{s}{e}^{-i\delta t}{S}^{z}/\hslash +{\tau }_{in}(t),$$

6 $${\partial }_{t}{f}_{M}=-(i{{\rm{\Delta }}}_{MF}+{\kappa }_{M}/\mathrm{2)}{f}_{M}+({\beta }_{1}+i{\beta }_{2}){S}^{-}+\hat{\xi }(t),$$

where Γ_1_ (Γ_2_) is the relaxation rate (dephasing rate) of the exciton, and *κ*_*M*_ is MFs decay rate. *τ*_*in*_(*t*) describe the Langevin noise follows the relations of $\langle {\tau }_{in}(t){\tau }_{in}^{\dagger }(t^{\prime} )\rangle  \sim \delta (t-t^{\prime} )$ and 〈*τ*_*in*_(*t*)〉 = 0. MFs are also influenced by stochastic force process with the following correlation function

7 $$\langle {\hat{\xi }}^{+}(t)\hat{\xi }(t^{\prime} )\rangle =\frac{{\kappa }_{M}}{{\omega }_{MF}}\int \frac{d\omega }{2\pi }\omega {e}^{-i\omega (t-t^{\prime} )}[1+\,\coth (\frac{\hslash \omega }{2{\kappa }_{B}T})],$$

where *k*_*B*_ is Boltzmann constant, *T* indicates the reservoir temperature. In Eq. (7), both the Brownian processes and non-Markovian processes^36^ will influence Majorana modes, and the quantum signatures of MFs modes can be observed only in the case of resolved sideband regime, i.e., *ω*_*MF*_/*κ*_*M*_ >> 1 under low temperature *T*. In the weak coupling regime, the effect of Brownian noise is modeled as Markovian processes. Further, the interaction between the QD and MFs is stronger than the interation with the reservoir, considering second order approximation^36^, then the reservoir that affects Majorana modes can be obtained as shown in Eq. (7).

### The Coherent Optical Response of QD

When the QD is driven by a strong pump laser, the Heisenberg operator can be divided into two parts, i.e., steady-state mean value *ρ*_0_, and small fluctuation *δρ* with zero mean value 〈*δρ*〉 = 0. To solve the steady-state values, we obtain the the steady-state population inversion (${\theta }_{0}=2{S}_{0}^{z}$) as follows

8 $$\begin{array}{c}{{\rm{\Gamma }}}_{1}({\theta }_{0}+\mathrm{1)[}{\theta }_{0}^{2}{({\beta }_{1}^{2}+{\beta }_{2}^{2})}^{2}-{\theta }_{0}({\beta }_{1}^{2}+{\beta }_{2}^{2})({{\rm{\Gamma }}}_{2}{\kappa }_{M}-2{{\rm{\Delta }}}_{c}{{\rm{\Delta }}}_{MF})\\ +({{\rm{\Delta }}}_{c}^{2}+{{\rm{\Gamma }}}_{2}^{2})({{\rm{\Delta }}}_{MF}^{2}+{\kappa }_{M}^{2}/\mathrm{4)]}+4{{\rm{\Omega }}}_{c}^{2}{\theta }_{0}{{\rm{\Gamma }}}_{2}({{\rm{\Delta }}}_{MF}^{2}+{\kappa }_{M}^{2}/\mathrm{4)}=0.\end{array}$$

We here make the ansatz to solve the fluctuation equation^33^ 〈*δρ*〉 = *ρ*_+_*e*^−*iδt*^ + *ρ*_−_*e*^*iδt*^, solving the fluctuation equation set, the linear optical susceptibility is ${\chi }_{eff}^{\mathrm{(1)}}({\omega }_{s})=\mu {S}_{+}({\omega }_{s})/{E}_{s}=({\mu }^{2}/\hslash {{\rm{\Gamma }}}_{2}){\chi }^{\mathrm{(1)}}({\omega }_{s})$, and *χ*^(1)^(*ω*_*s*_) is given by

9 $${\chi }^{\mathrm{(1)}}({\omega }_{s})=\frac{[{\varepsilon }_{7}{{\rm{\Pi }}}_{1}({{\rm{\Lambda }}}_{4}+{\varepsilon }_{3}{{\rm{\Pi }}}_{2})-i{\theta }_{0}{{\rm{\Lambda }}}_{4}]{{\rm{\Gamma }}}_{2}}{{{\rm{\Lambda }}}_{1}{{\rm{\Lambda }}}_{4}+{{\rm{\Pi }}}_{1}{{\rm{\Pi }}}_{2}{\epsilon }_{3}{\epsilon }_{4}},$$

where Π_1_ = 2[(*β*_1_ − *iβ*_2_)*f*_*M*0_ − *i*Ω_*c*_], Π_2_ = 2[(*β*_1_ + *iβ*_2_)*f*_*M*0_ + *i*Ω_*c*_], ϵ_1_ = (*β*_1_ + *iβ*_2_)/[*i*(Δ_*MF*_ − *δ*) + *κ*_*M*_/2], ϵ_2_ = (*β*_1_ + *iβ*_2_)/[*i*(Δ_*MF*_ + *δ*) + *κ*_*M*_/2], $${\epsilon }_{3}=[i{{\rm{\Omega }}}_{c}-({\beta }_{1}-i{\beta }_{2}){f}_{M0}-({\beta }_{1}+i{\beta }_{2}){S}_{0}{\epsilon }_{2}^{\ast }]/({{\rm{\Gamma }}}_{1}-i\delta )$$, $${\epsilon }_{4}=[i{{\rm{\Omega }}}_{c}+({\beta }_{1}+i{\beta }_{2}){f}_{M0}^{\ast }$$$$+({\beta }_{1}-i{\beta }_{2}){S}_{0}^{\ast }{\epsilon }_{1}]/({{\rm{\Gamma }}}_{1}-i\delta )$$, $${\epsilon }_{5}=[i{{\rm{\Omega }}}_{c}-({\beta }_{1}-i{\beta }_{2}){f}_{M0}-({\beta }_{1}+i{\beta }_{2}){S}_{0}{\epsilon }_{1}^{\ast }]/({{\rm{\Gamma }}}_{1}+i\delta )$$, $${\epsilon }_{6}=[i{{\rm{\Omega }}}_{c}+({\beta }_{1}+i{\beta }_{2}){f}_{M0}^{\ast }+({\beta }_{1}-i{\beta }_{2}){S}_{0}^{\ast }{\epsilon }_{2}]/({{\rm{\Gamma }}}_{1}+i\delta )$$, $${\epsilon }_{7}=i{S}_{0}^{\ast }/({{\rm{\Gamma }}}_{1}-i\delta )$$, ϵ_8_ = *iS*_0_/(Γ_1_ + *iδ*), Λ_1_ = *i*(Δ_*c*_ − *δ*) + Γ_2_ − *θ*_0_(*β*_1_ − *iβ*_2_)ϵ_1_ + Π_1_ϵ_4_, $${{\rm{\Lambda }}}_{2}=-i({{\rm{\Delta }}}_{c}-\delta )+{{\rm{\Gamma }}}_{2}-{\theta }_{0}({\beta }_{1}+i{\beta }_{2}){\epsilon }_{1}^{\ast }-{{\rm{\Pi }}}_{2}{\epsilon }_{5}$$, Λ_3_ = *i*(Δ_*c*_ + *δ*) + Γ_2_ − *θ*_0_(*β*_1_ − *iβ*_2_)ϵ_2_ + Π_1_ϵ_6_, Λ_4_ = −*i*(Δ_*c*_ + *δ*) + Γ_2_ − *θ*_0_(*β*_1_ + *iβ*_2_)ϵ_1_ + Π_2_ϵ_3_. The real and imaginary parts of *χ*^(1)^(*ω*_*s*_) show the dispersion and absorption, respectively. The nonlinear optical susceptibility can be calculated as $${\chi }_{eff}^{\mathrm{(3)}}({\omega }_{s})=\mu {S}_{-}({\omega }_{s})/\mathrm{(3}{E}_{c}^{2}{E}_{s})={\Sigma }_{3}{\chi }^{\mathrm{(3)}}({\omega }_{s})$$, where $${\Sigma }_{3}={\mu }^{4}/\mathrm{(3}{\hslash }^{3}{{\rm{\Gamma }}}_{2}^{3})$$, and *χ*^(3)^(*ω*_*s*_) is given by

10 $${\chi }^{\mathrm{(3)}}({\omega }_{s})=-\,\frac{{{\rm{\Pi }}}_{1}[{\epsilon }_{8}({{\rm{\Lambda }}}_{2}+{\epsilon }_{5}{{\rm{\Pi }}}_{2})-i{\theta }_{0}{\epsilon }_{5}]{{\rm{\Gamma }}}_{2}^{3}}{[{{\rm{\Lambda }}}_{2}{{\rm{\Lambda }}}_{3}+{{\rm{\Pi }}}_{1}{{\rm{\Pi }}}_{2}{\epsilon }_{5}{\epsilon }_{6}]{{\rm{\Omega }}}_{c}^{2}}.$$

The real and imaginary parts of *χ*^(3)^(*ω*_*s*_) characterize the Kerr coefficient and nonlinear absorption, respectively.

### The Numerical Results

We use the realistic parameters for the QD^37^: Γ_1_ = 0.3 GHz and Γ_2_ = Γ_1_/2. According to several recent experiment reports^3–7^, we expect that the decay rate *κ*_*M*_ = 0.1 × 10^6^ Hz. For the coupling strength *β* of the QD and MFs, we use the previous published work^38^, and the numerical value is *β* = 0.05 × 10^9^ Hz which is related to the distance between the QD and the hybrid S/SR device as shown in Fig. 1.

**Figure 2:**
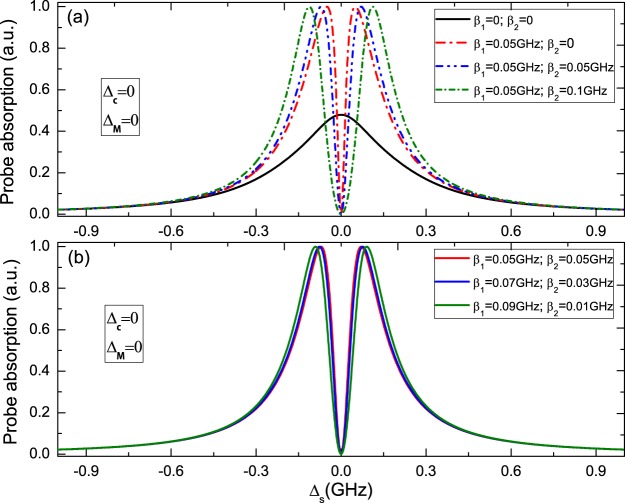
(**a**) and (**b**) The probe absorption spectra of probe field vs. the probe detuning Δ_*s*_ under ϵ_*MF*_ = 0 and Δ_*c*_ = 0 with several different QD-MFs coupling strengths. The parameters used are Γ_1_ = 0.3 GHz, Γ_2_ = 0.15 GHz, *κ*_*M*_ = 0.1 MHz, ${{\rm{\Omega }}}_{c}^{2}=0.005$(GHz)^2^.

Firstly, we consider uncoupled majorana modes (i.e. ϵ_*MF*_ = 0), that is to say the two MFs are not coupled to each other and probe field is resonant with the exciton frequency (i.e. Δ_*c*_ = 0). In this situation, the Hamiltonian of the coupled QD-MFs can be reduced to ${H}_{QD-MFs}=i\hslash {\beta }_{1}({S}^{-}{f}_{M}^{\dagger }-{S}^{+}{f}_{M})-\hslash {\beta }_{2}({S}^{-}{f}_{M}^{\dagger }+{S}^{+}{f}_{M})$, which is similar to the J-C Hamiltonian in quantum optics. Figure 2(a) displays the probe absorption spectra of the probe laser (i.e., the imaginary part of the dimensionless susceptibility Im *χ*^(1)^) *vs*. the detuning Δ_*s*_ (Δ_*s*_ = *ω*_*s*_ − *ω*_*e*_) under several different QD-MFs coupling strengths. The black solid curve in Fig. 2(a) shows the result of no QD-MFs coupling, which present Lorentz line shape under weak pump laser driven. When a pair of MFs emerge in the end of the hybrid S/SR device and interact with the two-level QD, the absorption spectrum presents a symmetric Rabi splitting when *β*_1_ = 0.05 × 10^9^ Hz and *β*_2_ = 0 as shown the red curve in Fig. 2(a). Thus, the symmetric Rabi splitting in the probe absorption reveals a true signature of MFs emerge in the hybrid S/SR device. To determine the symmetric Rabi splitting of the QD is induced by MFs modes rather than other regimes induced the Majorana-like signatures, we consider several conditions. Due to Kondo effect can also leads to analogous Majorana signatures, it is necessary to distinguish the true Majorana signatures and Kondo effect. Kondo effect, in the detection of MFs with electrical methods, is generally related to the strong coupling to two normal leads in he hybrid S/SR structure, and when the Kondo temperature *T*_*k*_ is smaller than the gap, a superconducting gap can suppress the effect. Recently, to control Kondo effect, Nadj-Perge *et al*. have reported an experimental project of the hybrid Fe-atoms-chain/Pb-superconductor (Fe/Pb) substrate^6^, and the present another evidence in the scheme is related to superconductivity and rather than with other phenomena. Therefore, in a way, the Fe/Pb substrate can replace the hybrid S/SR structure. On the other hand, due to there are several normal electrons in semiconductor nanowire ring, so it is necessary to determine whether the signals in the probe absorption spectra are true QD-MFs coupling, rather than the interation between the QD and the electrons in the nanowire induced the same Majorana signals. For this question, we once have used the tight-binding Hamiltonian^34^ to elucidate the electrons in nanowire, and the numerical results indicate MFs signatures in the coherent optical spectra are the true MFs signals^34^.

When a pair of MFs couple to the QD (*β*_1_ = 0.05 GHz, *β*_2_ = 0.05 GHz), the peak width of Rabi splitting induced by MFs is enhanced significantly (the blue curve in Fig. 2(a)), and the bigger QD-MFs coupling induced larger peak width of splitting as green curve in Fig. 2(a). In Fig. 2(b), we also show three different QD-MFs coupling strength, i.e. (*β*_1_ = 0.05 GHz, *β*_2_ = 0.05 GHz), (*β*_1_ = 0.07 GHz, *β*_2_ = 0.03 GHz), and (*β*_1_ = 0.09 GHz, *β*_2_ = 0.01 GHz). The results indicate that the peak width of Rabi splitting depends on one of the pair of MFs coupling to the QD. The physical origin of such results are due to the coherent interaction between the QD and MFs, and we introduce the dressed-state theory to describe the interation between the QD and MFs for interpreting this physical phenomena. Due to the QD is considered as a TLS with the ground state $|0\rangle$ and exciton state $|1\rangle$, when QD couple to MFs, the two-level QD will be embellish by MFs number states *n*_*MF*_ and generate four Majorana dressed states, i.e., $|\mathrm{0,}\,{n}_{MF}\rangle$, $|\mathrm{0,}\,{n}_{MF}+1\rangle$, $|\mathrm{1,}\,{n}_{MF}\rangle$, $|\mathrm{1,}\,{n}_{MF}+1\rangle$, where *n*_*MF*_ indicates MFs number states as shown in Fig. 1(c). Then, in Fig. 2, the left sharp peaks of Rabi splitting shows the transition from $|0\rangle$ to $|\mathrm{1,}\,{n}_{MF}\rangle$, and the right one gives the transition between $|0\rangle$ and $|\mathrm{1,}\,{n}_{MF}+1\rangle$.

**Figure 3:**
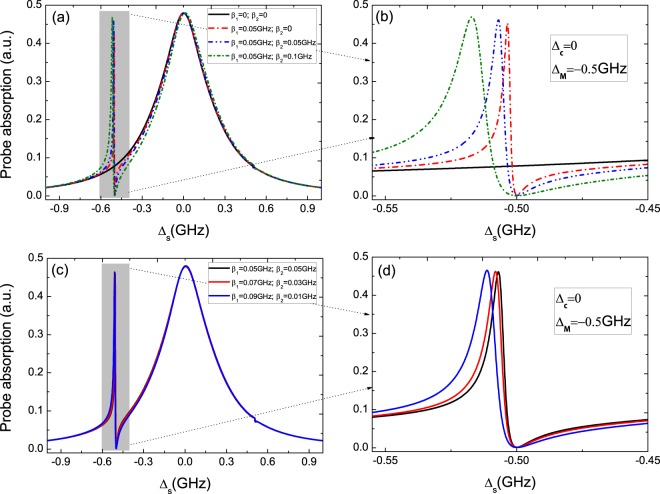
(**a**) The probe absorption spectra at coupled majorana modes (ϵ_*MF*_ ≠ 0) and Δ_*c*_ = 0. (**b**) The detail parts of the left peaks in (**a**). (**c**) The probe absorption spectra in the condition of a pair MFs coupled the QD. (**d**) The detail parts of the left peaks in (**c**).

Secondly, we consider coupled Majorana modes (ϵ_*MF*_ ≠ 0) and Δ_*c*_ = 0 as shown in Fig. 3. In this condition, the interaction between the two MFs should be taken into consideration. As in uncoupled Majorana modes, if there is no QD-MFs coupling, the spectrum (the black curve in Fig. 3(a)) shows the same Lorentz line shape. When a pair of MFs coupled to the QD, the spectra display unsymmetric Rabi splitting under the detuning Δ_*MF*_ = −0.5 × 10^9^ Hz. Figure 3(b) is the detail parts of the left peaks in Fig. 3(a), and sharp peak locates at Δ_*s*_ = −0.5 GHz which is different from in uncoupled majorana modes. In Fig. 3(c), we further discuss several different QD-MFs coupling strength, the results in probe absorption spectra manifest that when the QD is close to one MF (such as *γ*_*M*1_, and then *β*_1_ > *β*_2_), the width of unsymmetric Rabi splitting is larger than the case of *β*_1_ = *β*_2_, and Fig. 3(d) is the detail parts of the left peaks in Fig. 3(c). The QD-MFs coupling strength can be controlled with controlling the distance of the QD and the hybrid S/SR device. The results in Fig. 2 and Fig. 3 manifest that the signals are the ture MFs signature, and our all-optical detection means can work at the coupled- and uncoupled-Majorana edge states^38^.

In addition, when making a comparison between ϵ_*MF*_ ≠ 0 in Fig. 3 and ϵ_*MF*_ = 0 in Fig. 2, we find that the probe absorption spectra show the analogous phenomenon of electromagnetically induced transparency (EIT)^39^ in the two situations. The absorption dip approaches zero at Δ_*s*_ = 0 and Δ_*s*_ = −0.5 × 10^9^ Hz under ϵ_*MF*_ = 0 and ϵ_*MF*_ ≠ 0, respectively, which indicates the probe field is transmitted with experiencing nothing absorption. The result is due to the destructive quantum interference effect between the beat frequency *δ* (*δ* = *ω*_*s*_ − *ω*_*c*_) of the two optical fields (i.e. pump laser field and probe laser field) via the QD and Majorana modes^38^. When the beat frequency *δ* approximates to the MFs resonance frequency *ω*_*MF*_, then Majorana modes start oscillating coherently resulting in Stokes-like (Δ_*SM*_ = *ω*_*c*_ − *ω*_*MF*_) and anti-Stokes-like (Δ_*ASM*_ = *ω*_*c*_ + *ω*_*MF*_) light scattering from the QD. In highly off-resonant, the process of Δ_*SM*_ is strongly suppressed, and only leaving the process of Δ_*ASM*_ interferes with the probe field modifying the absorption spectra. We therefore define the phenomenon as Majorana modes induced transparency (MMIT)^38^, which will induce remarkable phenomena of slow light.

**Figure 4:**
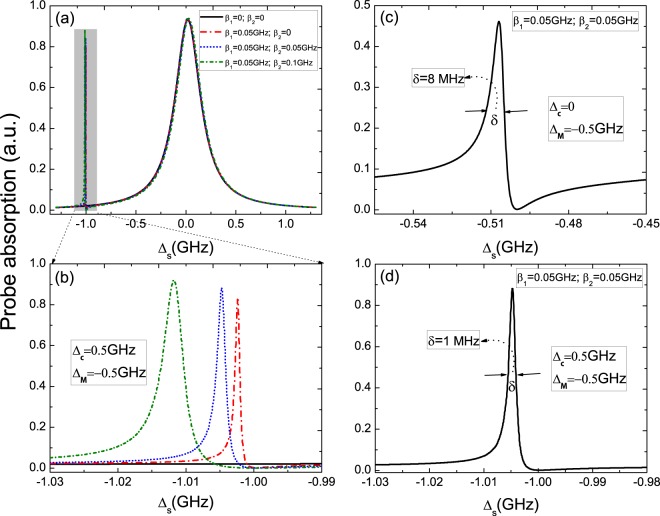
(**a**) The probe absorption spectra at Δ_*MF*_ = −0.5 GHz, Δ_*c*_ = 0.5 GHz, and (**b**) is the detail parts of the left peaks in (**a**). (**c**) The probe absorption spectrum shows a bandwidth about 8.0 MHz at Δ_*c*_ = 0 and Δ_*MF*_ = −0.5 GHz. (**d**) The absorption spectrum shows a bandwidth about 1.0 MHz at Δ_*c*_ = 0.5 GHz and Δ_*MF*_ = −0.5 GHz.

Thirdly, we further consider coupled majorana modes (ϵ_*MF*_ ≠ 0) but adjust the detuning Δ_*c*_ from Δ_*c*_ = 0 to Δ_*c*_ = 0.5 × 10^9^ Hz. In this case, the location of the left peaks of unsymmetric Rabi splitting coincides with frequency shift induced by the exciton-pump field detuning, and then the resonant makes the coherent interaction between the QD and a couple of MFs more stronger. Figure 4(a) gives the absorption spectra with several different QD-MFs coupling strength under two detuning, i.e. Δ_*MF*_ = −0.5 × 10^9^ Hz, Δ_*c*_ = 0.5 × 10^9^ Hz, which shows more remarkable unsymmetric splitting and the intensity is also enhanced simultaneously. Figure 4(b) is the detail parts of the left peaks in Fig. 4(a). Moreover, we make a comparison between the two situations, i.e. (Δ_*c*_ = 0, Δ_*MF*_ = −0.5 × 10^9^ Hz) and (Δ_*c*_ = 0.5 × 10^9^ Hz, Δ_*MF*_ = −0.5 × 10^9^ Hz) at a pair of coupling strength *β*_1_ = 0.05 × 10^9^ Hz and *β*_2_ = 0.05 × 10^9^ Hz. The probe absorption spectrum shows that the bandwidth is about 8 MHz and the intensity is about 4.5 with the left sharp peaks exactly locating at Δ_*s*_ = −0.5 × 10^9^ Hz under the condition of Δ_*c*_ = 0 and Δ_*MF*_ = −0.5 × 10^9^ Hz as shown in Fig. 4(c). However, when adjusting the detuning Δ_*c*_ from Δ_*c*_ = 0 to Δ_*c*_ = 0.5 × 10^9^ Hz, one can obtain the narrower bandwidth (about 1 MHz) and the stronger intensity (about 0.9) locating at Δ_*s*_ = −1.0 × 10^9^ Hz as shown in Fig. 4(d). In this case, the sideband peak induced by coupled majorana modes coincides with sharp peaks induced by pump off-resonant Δ_*c*_ = 0.5 × 10^9^ Hz, which makes the coherent interaction of QD-MF more strong.

**Figure 5:**
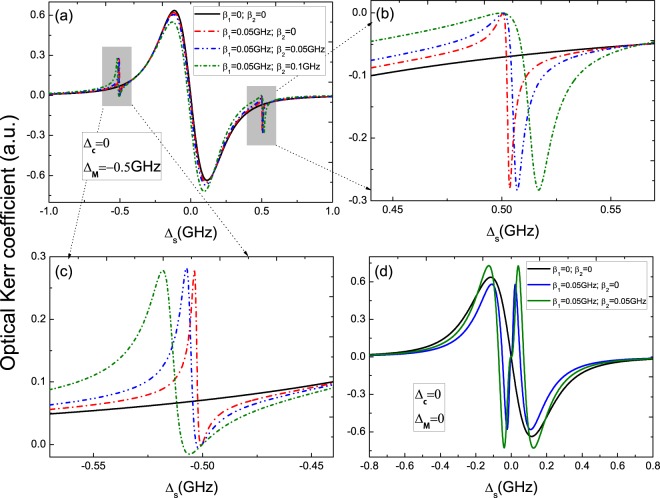
(**a**) The optical Kerr coefficient as a function of Δ_*s*_ at coupled majorana modes (ϵ_*MF*_ ≠ 0) and the detuning Δ_*c*_ = 0. (**b**) and (**c**) are the detail parts of the right and left peaks in (**a**). (**d**) The optical Kerr coefficient at ϵ_*MF*_ = 0 and Δ_*c*_ = 0.

Moreover, we slao investigate the optical Kerr nonlinear effect of QD mediated by MFs. Figure 5(a) gives the optical Kerr coefficient (*Re*(*χ*^(3)^)) as a function of Δ_*s*_ under ϵ_*MF*_ ≠ 0 with the resonance detuning Δ_*c*_ = 0. In Fig. 5(a), the black curve displays the nonlinear optical Kerr spectrum without the QD-MFs coupling, the red curve gives the QD couples to one MFs with coupling strength *β*_1_ = 0.05 GHz, the blue one is that the QD couple to a pair of MFs with coupling strength *β*_1_ = 0.05 GHz and *β*_2_ = 0.05 GHz under Δ_*c*_ = 0 and Δ_*MF*_ = −0.5 GHz. Figure 5(b,c) are the detail parts of the right and left peaks in Fig. 5(a). It is obvious that when the MFs emerge in the hybrid S/SR device and couple to the QD, two sharp sideband peaks will emerge in the optical Kerr spectra. The physical origin of the results are due to the coherent interaction between the QD and MFs, which makes the resonant enhancement of the optical Kerr effect in the QD. Figure 5(d) shows optical Kerr coefficient at uncoupled Majorana modes with no QD-MFs coupling, one QD-MFs coupling, and a pair of QD-MFs coupling, optical Kerr spectra also present splitting when the QD couple to MFs. The results signify that the sharp peaks in the nonlinear optical spectra may be the signature of real MFs which gives the nonlinear optical means to detect MFs.

**Figure 6:**
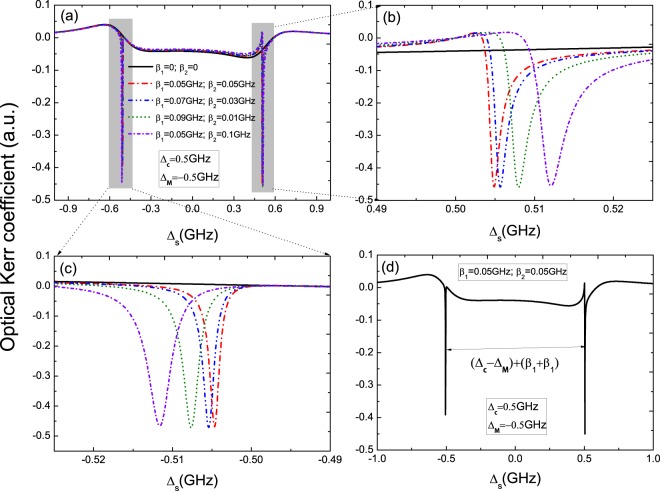
(**a**) The optical Kerr coefficient at ϵ_*MF*_ ≠ 0 and Δ_*c*_ = 0.5 GHz with several different QD-MFs coupling strength, (**b**) and (**c**) are the details in (**a**). (**d**) The width of splitting in the optical Kerr spectrum is determined by Δ_*c*_, Δ_*MF*_ and QD-MFs coupling strength *β*_2_ and *β*_2_.

Switching Δ_*c*_ from Δ_*c*_ = 0 to Δ_*c*_ = 0.5 GHz, Fig. 6(a) presents the optical Kerr coefficient vs. the detuning Δ_*s*_ at coupled Majorana modes under several different QD-MFs coupling strengths, and the details are shown in Fig. 6(b,c). It is obvious that the width of splitting induced by a pair of MFs couple to the QD is larger than in the case of one MF couple to the QD. Furthermore, the width of splitting in the optical Kerr spectrum is determined by the two detuning and QD-MFs coupling strength *β*_2_ and *β*_2_ as shown in Fig. 6(d).

## Discussion

We have designed a hybrid S/SR device and introduced an optical pump-probe technology to probe MFs with QDs which is very different from the current electrical method. In the previous theoretical schemes for detecting MFs with QD, QD is considered as only one resonant level with single spin state and they only consider one adjacent MF interact with the QD. In this work, we investigate a pair of MFs coupled to the QD which induces remarkable splitting in the probe absorption spectrum and nolinear optical Kerr spectrum. The symmetric and unsymmetric Rabi splitting and optical Kerr nonlinear effect of QD mediated by MFs are studied under uncoupled and coupled majorana modes, respectively. Due to QD-MFs coupling, the probe absorption spectra present the phenomenon of Majorana modes induced transparency which will induce remarkable phenomena of slow light. With adjust the exciton resonant detuning and Majorana resonant detuning, the probe absorption intensity and the peak width of splitting are enhanced significantly, and the peak width of splitting in the optical Kerr spectrum depends on the coupling strength and resonant detuning.

Compared with electrical measurement, the optical scheme for the detection of MFs have several advantage, the first one is that there is no contact between the QD and the hybrid S/SR device, thus the introduce of noises can be avoided effectively, and finally the sensitivity of the measurement will enhance observably. The second one is that nanostructures such as QD have obtained remarkable progress in modern nanoscience, which paves a way to detect MFs experimentally. Therefore, the optical detection of MFs with QD is scientific and feasible based on recent experiment. The scheme proposed here may provide potential applications in quantum computing based on MFs with optically controlled in solid-state systems.

## Methods

The Heisenberg operator (*ρ* = *S*^*z*^, *S*^−^, *f*_*M*_) can be divided into two parts including steady-state mean value *ρ*_0_ and small fluctuation *δρ*, i.e. ${S}^{z}={S}_{0}^{z}+\delta {S}^{z}$, *S*^−^ = *S*_0_ + *δS*^−^, *f*_*M*_ = *f*_*M*0_ + *δf*_*M*_. Inseting these operators into Eqs (4–6), we then abtain the following equations

11 $${\partial }_{t}{S}^{z}=-{{\rm{\Gamma }}}_{1}[{S}_{0}^{z}+\delta {S}^{z}+1/\mathrm{2]}-({\beta }_{1}+i{\beta }_{2})({S}_{0}+\delta {S}^{-})({f}_{M0}^{\ast }+\delta {f}_{M}^{+})-({\beta }_{1}-i{\beta }_{2})({S}_{0}^{\ast }+\delta {S}^{+})({f}_{M0}+\delta {f}_{M})+i{{\rm{\Omega }}}_{c}[({S}_{0}^{\ast }+\delta {S}^{+})-({S}_{0}+\delta {S}^{-})]+\frac{i\mu {E}_{s}}{\hslash }({S}_{0}^{\ast }{e}^{-i\delta t}+\delta {S}^{+}{e}^{-i\delta t}-{S}_{0}{e}^{i\delta t}-\delta {S}^{-}{e}^{i\delta t}),$$

12 $${\partial }_{t}{S}^{-}=-(i{{\rm{\Delta }}}_{c}+{{\rm{\Gamma }}}_{2})({S}_{0}+\delta {S}^{-})+\mathrm{2(}{\beta }_{1}-i{\beta }_{2})({S}_{0}^{z}+\delta {S}^{z})({f}_{M0}+\delta {f}_{M})-2i{{\rm{\Omega }}}_{c}({S}_{0}^{z}+\delta {S}^{z})-\frac{2i\mu {E}_{s}}{\hslash }{e}^{-i\delta t}({S}_{0}^{z}+\delta {S}^{z})+\hat{\tau }(t),$$

13 $${\partial }_{t}{f}_{M}=-(i{{\rm{\Delta }}}_{MF}+{\kappa }_{M}/\mathrm{2)(}{f}_{M0}+\delta {f}_{M})+({\beta }_{1}+i{\beta }_{2})({S}_{0}+\delta {S}^{-})+\hat{\xi }(t),$$

To solve Eqs (11–13), we get two equation sets. The first one is steady-state mean equation set as follows

14 $${{\rm{\Gamma }}}_{1}({S}_{0}^{z}+1/\mathrm{2)}+({\beta }_{1}+i{\beta }_{2}){S}_{0}{f}_{M0}^{\ast }+({\beta }_{1}-i{\beta }_{2}){S}_{0}^{\ast }{f}_{M0}=i{{\rm{\Omega }}}_{c}({S}_{0}^{\ast }-{S}_{0}),$$

15 $$(i{{\rm{\Delta }}}_{c}+{{\rm{\Gamma }}}_{2}){S}_{0}+\mathrm{2(}{\beta }_{1}-i{\beta }_{2}){S}_{0}^{z}{f}_{M0}=2i{{\rm{\Omega }}}_{c}{S}_{0}^{z},$$

16 $$(i{{\rm{\Delta }}}_{MF}+{\kappa }_{M}/\mathrm{2)}{f}_{M0}-({\beta }_{1}+i{\beta }_{2}){S}_{0}=0,$$

which determines Eq. (8). The second one is the fluctuation equation set which is

17 $$\begin{array}{ccc}\delta {\dot{S}}^{z} & = & -\,{{\rm{\Gamma }}}_{1}\delta {S}^{z}-({\beta }_{1}+i{\beta }_{2})({S}_{0}\delta {f}_{M}^{+}+{f}_{M0}^{\ast }\delta {S}^{-})-({\beta }_{1}-i{\beta }_{2})({S}_{0}^{\ast }\delta {f}_{M}+{f}_{M0}\delta {S}^{+})\\  &  & +i{{\rm{\Omega }}}_{c}(\delta {S}^{+}-\delta {S}^{-})+\frac{i\mu {E}_{s}}{\hslash }[{S}_{0}^{\ast }{e}^{-i\delta t}-{S}_{0}{e}^{i\delta t}],\end{array}$$

18 $$\delta {\dot{S}}^{-}\,=\,-\,(i{{\rm{\Delta }}}_{c}\,+\,{{\rm{\Gamma }}}_{2})\delta {S}^{-}\,+\,{\theta }_{0}({\beta }_{1}\,-\,i{\beta }_{2})\delta {f}_{M}\,+\,\mathrm{2[}{f}_{M0}({\beta }_{1}\,-\,i{\beta }_{2})\,-\,i{{\rm{\Omega }}}_{c}]\delta {S}^{z}\,-\,\frac{i\mu {w}_{0}{E}_{s}}{\hslash }{e}^{-i\delta t},$$

19 $$\delta {\dot{f}}_{M}=-(i{{\rm{\Delta }}}_{MF}+{\kappa }_{M}/\mathrm{2)}\delta {f}_{M}+({\beta }_{1}+i{\beta }_{2})\delta {S}^{-},$$

Solving Eqs (17–19), we obtain Eqs (9 and 10).
